# Intragenomic variations of multicopy ITS2 marker in *Agrodiaetus* blue butterflies (Lepidoptera, Lycaenidae)

**DOI:** 10.3897/CompCytogen.v9i4.5429

**Published:** 2015-08-07

**Authors:** Nazar A. Shapoval, Vladimir A. Lukhtanov

**Affiliations:** 1 Department of Karyosystematics, Zoological Institute of Russian Academy of Sciences, Universitetskaya nab. 1, St. Petersburg 199034, Russia; 2 Department of Entomology, Faculty of Biology, St. Petersburg State University, Universitetskaya nab. 7/9, St. Petersburg 199034, Russia

**Keywords:** *Agrodiaetus*, Lycaenidae, *Polyommatus*, *ITS2*, ribosomal DNA, intraindividual variability, cloning

## Abstract

The eukaryotic ribosomal DNA cluster consists of multiple copies of three genes, *18S*, 5. *8S* and *28S rRNAs*, separated by multiple copies of two internal transcribed spacers, *ITS1* and *ITS2*. It is an important, frequently used marker in both molecular cytogenetic and molecular phylogenetic studies. Despite this, little is known about intragenomic variations within the copies of eukaryotic ribosomal DNA genes and spacers. Here we present data on intraindividual variations of *ITS2* spacer in three species of *Agrodiaetus* Hübner, 1822 blue butterflies revealed by cloning technique. We demonstrate that a distinctly different intragenomic *ITS2* pattern exists for every individual analysed. *ITS2* sequences of these species show significant intragenomic variation (up to 3.68% divergence), setting them apart from each other on inferred phylogenetic tree. This variation is enough to obscure phylogenetic relationships at the species level.

## Introduction

The eukaryotic ribosomal DNA (rDNA) cluster consists of three genes, *18S*, 5. *8S* and *28S rRNAs*, separated by two internal transcribed spacers, *ITS1* and *ITS2*. This array forms a transcription unit, which is are typically represented in a genome by several hundred tandemly repeated copies (Long and David 1980, Gerbi 1985). The number of rDNA sequence variants can vary within a wide range both at the species and individual level. For example, different species of *Drosophila* Linnaeus, 1758 are estimated to have three to 18 variants of rDNA sequences (Stage and Eickbush 2007). The genome of sea sponge *Amphimedon queenslandica* Hooper & van Soest, 2006 was found to contain approximately 14.5 copies of rDNA sequences per haploid complement (Srivastava et al. 2010). Furthermore, individuals of the same species can have very different numbers of rDNA copies because the clusters display both meiotic rearrangements and somatic mosaicism. It has been shown that in humans for example, the number of rDNA sequences even within a single cluster can vary in an enormous extent, from one repeat unit up to 140 repeats (Stults et al. 2008).

Ribosomal RNA genes have been widely used in taxonomy, biogeographic, phylogenetic analyses, and molecular cytogenetic studies (Hillis and Davis 1986, Mindell and Honeycutt 1990, Wesson et al. 1993, Vogler and DeSalle 1994). In particular, more detailed and precise karyotypes studies became available since fluorescence *in situ* hybridization (FISH) technology was applied to the chromosomal physical mapping. FISH mapping identifies useful chromosomal markers that can be applied to studies of genome organization and species evolution and can also identify specific chromosomes, homologous chromosomes, chromosome rearrangements and sex chromosomes, among others (Nakajima et al. 2012). Ribosomal RNA genes are among the most mapped sequences in chromosomes in many animal groups including insects (Cabrero and Camacho 2008, Grozeva et al. 2010, 2011, Nguyen et al. 2010, Kuznetsova et al. 2012, Maryańska-Nadachowska et al. 2013, Gokhman et al. 2014, Kuznetsova et al. 2015, Vershinina et al. 2015).

Accordingly, rDNA can be excellent source of cytogenetic markers for comparative genomic studies, evolutionary studies as well as the genetic identification of species (Mantovani et al. 2005, Pedrosa-Harand et al. 2006, Cabral-de-Mello et al. 2011).

At the nucleotide sequence level coding regions and spacers can reveal phylogenetic relationships ranging from the level of major phyla of living organisms to the population level, because they differ widely in their rate of evolution (Hillis and Dixon 1991, Wesson et al. 1992, Kuperus and Chapco 1994, Muccio et al. 2000, Wiegmann et al. 2000). *18S* and *28S* rDNA genes are reported to be highly informative to reconstruct higher-level phylogenies in plants and animals (see e.g. Soltis et al. 2000, Mukha et al. 2002).

Unlike highly conserved rRNA genes, non-coding fast evolving transcribed spacers have high level of interspecific variability. Therefore, the internal transcribed spacers are considered to be useful phylogenetic markers, specifically for low-level phylogenetic analyses. *ITS1* and *ITS2* have been used extensively in phylogenetic reconstruction of closely related species and cryptic species complexes (Wilkerson et al. 2004). For instance, *ITS* have become the standard barcode of choice in most investigations for plants and fungi (Stoeckle 2003, Kress et al. 2005, Sass et al. 2007, Bellemain et al. 2010, Hollingsworth et al. 2011, Schocha et al. 2012, Li et al. 2015).

During PCR all variants of *ITS* sequences presented in genome are amplified, therefore, direct sequencing could lead to inaccurate or erroneous phylogenetic reconstructions. Accordingly identifying and examination levels of intragenomic and intraspecific variation among *ITS* sequences are of real importance.

Agrodiaetus is a species-rich subgenus within the Palearctic genus *Polyommatus* (Talavera et al. 2013). The subgenus includes ca. 130 described species (see Vila et al. 2010, Lukhtanov et al. 2008, 2015a, Vershinina and Lukhtanov 2010, Przybyłowicz et al. 2014, Lukhtanov and Tikhonov 2015). The subgenus was estimated to have originated only about three million years ago (Kandul et al. 2004). Nowadays this rapidly radiated group of butterflies is a model system in studies of speciation (Lukhtanov et al. 2005, Lukhtanov et al. 2015b), and rapid karyotype evolution (Kandul et al. 2007). Several molecular phylogenetic studies have been conducted on *Agrodiaetus*, also based on *ITS2* molecular marker (Wiemers 2003, Wiemers et al. 2009, Wiemers et al. 2010, Lukhtanov et al. 2015a). However, until now rate of *ITS2* intragenomic variations in this rapidly evolved group have never been analyzed.

This paper addresses a more detailed analysis of intraindividual variability of *ITS2* region in three Polyommatus (Agrodiaetus) species: Polyommatus (Agrodiaetus) peilei Bethune-Baker, 1921, Polyommatus (Agrodiaetus) karindus (Reiley, 1921) and Polyommatus (Agrodiaetus) morgani (Le Cerf, 1909). These three species are closely related to each other (Lukhtanov et al. 2015b), but have clear differences in male wing color and karyotypes (haploid chromosome number are n= 38-39 in Polyommatus (Agrodiaetus) peilei; n=68 and n=73 in different populations of Polyommatus (Agrodiaetus) karindus; and n=25-27 in Polyommatus (Agrodiaetus) morgani) (Lukhtanov et al. 2015b). Direct sequencing of *ITS2* give ambiguous results; thus, we sought to clone and sequence *ITS2* from these species to quantify the prevalence of intragenomic *ITS2* variation and determine its effect on phylogenetic reconstructions.

## Material and methods

Butterflies (only males) were collected in NW Iran (Zagros mt., Kordestan provience) in 2007–2014. Bodies were placed in 2 ml plastic vials with 100% ethanol for DNA analysis. Wings were stored in glassine envelopes for morphological study. All samples are stored at Zoological Institute, St Petersburg, Russia.

*ITS2* region was amplified using the primer pair: ITS-3 and ITS-4 (White et al. 1990). When ITS-3 and ITS-4 primers failed to amplify a sufficient product, self-designed lepidopteran primers were used:

ILYC2F 5`- GAGAAACATCCAGGACCACT - 3` and

ILYC2RB 5` - CTGATCTGAGGCCA ACG - 3`.

The PCR amplifications were performed either in 50 µl reaction volume containing ca. 10–20 ng genomic DNA and 0.5 mM of each primer, using 26 PCR Master Mix (Fermentas, Lithuania). The temperature profile was as follows: initial denaturation at 94 °C for 1 min, followed by 30 cycles of denaturation at 94 °C for 45 s, annealing at 50 °C for 45 s, and extension at 72 °C for 1 min with a final extension at 72 °C for 10 min.

Amplified fragments were purified using GeneJET Gel Extraction Kit (Fermentas, Lithuania). Purification was carried out according to the manufacturer’s protocol. The success of PCR amplification and purification was evaluated by electrophoresis of the products in 1% agarose gel. Purified PCR product was used for direct sequencing or subsequent cloning.

*ITS2* PCR products were cloned into blunt-end cloning vector pJET1.2 (Fermentas, Lithuania) according to the manufacturer’s protocol for 10 minutes at room temperature. The pJet1.2 plasmid selects successful ligations through the disruption of an otherwise lethal gene, eco47IR, which enables positive selection of the recombinants. Before ligation, a 3’-A overhang were removed from the PCR products by treating the PCR product with a proofreading DNA polymerase. For transformation 5 µl of the ligation mixture reaction were added to 50 µl of chemo-competent *Escherichia coli* DH101B cells an incubated for 10 min. on ice. After incubation transformation mixture were pipetted onto pre-warmed LB Anp IPTG agar plate and spread by using inoculation loop. Agar plates with competent *Escherichia coli* were incubated overnight at 37 °C.

For each cloning, more than 500 clones were obtained. To check if the cloning procedures were successful, PCR with *ITS2*-speciffic primers were conducted for 20 colonies per cloning reaction. GeneJET Plasmid Miniprep Kit (Fermentas, Lithuania) was used for preparation of plasmid DNA from recombinant *Escherichia coli* culture. A single colony from a freshly streaked selective plate were picked to inoculate 1–5 mL of LB medium supplemented with ampicillin and incubated for 12–16 hours at 37 °C while shaking at 200–250 rpm. The bacterial culture was harvested by centrifugation at 8000 rpm (6800 × g) in a microcentrifuge for 2 min at room temperature. The supernatant was decanted and all remaining medium was removed. The pelleted cells were resuspended and subjected to SDS/alkaline lysis to liberate the plasmid DNA. The resulting lysate was neutralized to create appropriate conditions for binding of plasmid DNA on the silica membrane in the spin column. Cell debris and SDS precipitate were pelleted by centrifugation, and the plasmid DNA were washed to remove contaminants and eluted.

Sequencing was carried out using 3500xL analyzer (Applied Biosystems). Not less than 300 ng of plasmid DNA template was used for sequencing procedure. Cloned fragments were analyzed edited and aligned in Bioedit Software.

A Bayesian approach for estimating phylogeny was used. Bayesian trees were inferred using partitioned models: GTR for nucleotide substitutions and standard model for indels as implemented in MRBAYES v. 3.2 (Ronquist and Huelsenbeck 2012). Each gap (indel) was treated as a single character regardless of the length of the gap, under the assumption that a given gap is a result from one mutational event (Simmons and Ochoterena 2000).

## Results

**Figure 1.**
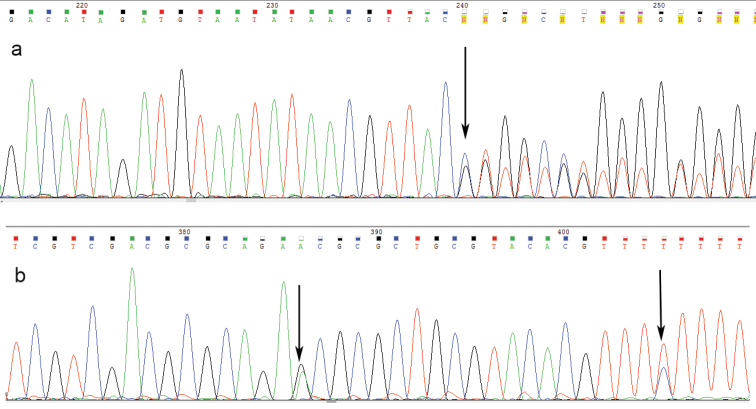
Examples of results from direct sequencing of *ITS2*. **a** Example of polymorphism caused by an indel (black arrow indicate the beginning position of an indel) **b** Example of single nucleotide substitutions (indicated by black arrows).

Sequenced region contained 3` end of *5.8S* gene, *ITS2*, and the 5` end of the *28S* gene. Direct sequencing of amplicons of 30 individuals (10 individuals per each species) displayed intra-individual heterogeneities in all specimens analyzed. There are two kinds of heterogeneities: single nucleotide substitutions and mono, bi- and multi-nucleotide insertions/deletions. The presence of heterogeneities was indicated by double peaks in substitution positions, and by a series of mixed peaks in case of indel events, both positioned after a sequence of good quality. The examples of heterogeneities revealed by direct sequencing are displayed in Figure 1.

To elucidate the visible heterogeneity, the amplicons for 2 specimens of Polyommatus (Agrodiaetus) peilei, 2 specimens of Polyommatus (Agrodiaetus) karindus and one specimen of Polyommatus (Agrodiaetus) morgani were cloned and 10 clones per specimen were sequenced. The summary of the heterogeneities in the *ITS* region displayed by the clones is depicted in Table 1. Partial sequences of *5,8S* and *28S* genes were cropped from further analysis. Total length of *ITS2* varied from 477 bp up to 512 bp depending on the presence of insertions\deletions. Uncorrected “p” pairwise distances for all clones are given in Table 2.

**Table 1.** Variable positions among sequenced clones.

Specimen	Clone number	Position																																									
		**128**	130	**131**	171	172	173	235	316	**326**	329	330	331	332	333	334	335	**336**	**337**	338	339	340	341	342	343	344	**345**	346	356	400	414	465											
W136 *Polyommatus (Agrodiaetus) peilei*	#01	**T**	**G**	**-**	**C**	**G**	**C**	**A**	**A**	**T**	**T**	**T**	**T**	**T**	**T**	**T**	**T**	**-**	**-**	**C**	**G**	**T**	**T**	**T**	**T**	**T**	**-**	**C**	**G**	**G**	**G**	**C**											
W136 *Polyommatus (Agrodiaetus) peilei*	#02	**T**	**G**	**-**	**C**	**G**	**C**	**A**	**A**	**T**	**T**	**T**	**T**	**T**	**T**	**T**	**T**	**-**	**-**	**C**	**G**	**T**	**T**	**T**	**T**	**T**	**-**	**C**	**G**	**G**	**G**	**T**											
W136 *Polyommatus (Agrodiaetus) peilei*	#03	**T**	**A**	**A**	**C**	**A**	**C**	**G**	**G**	**C**	**T**	**T**	**T**	**T**	**T**	**T**	**T**	**T**	**T**	**T**	**G**	**T**	**T**	**T**	**T**	**T**	**-**	**C**	**A**	**G**	**A**	**C**											
W136 *Polyommatus (Agrodiaetus) peilei*	#04	**T**	**G**	**-**	**C**	**G**	**C**	**A**	**A**	**T**	**T**	**T**	**T**	**T**	**T**	**T**	**T**	**-**	**-**	**T**	**G**	**T**	**T**	**T**	**T**	**T**	**-**	**C**	**G**	**G**	**G**	**C**											
W136 *Polyommatus (Agrodiaetus) peilei*	#05	**T**	**A**	**A**	**C**	**A**	**C**	**G**	**G**	**C**	**T**	**T**	**T**	**T**	**T**	**T**	**T**	**T**	**T**	**T**	**G**	**T**	**T**	**T**	**T**	**T**	**T**	**-**	**A**	**G**	**G**	**C**											
W136 *Polyommatus (Agrodiaetus) peilei*	#06	**T**	**G**	**-**	**C**	**G**	**C**	**A**	**A**	**T**	**T**	**T**	**T**	**T**	**T**	**-**	**-**	**-**	**-**	**T**	**G**	**T**	**T**	**T**	**T**	**T**	**-**	**C**	**G**	**G**	**G**	**C**											
W136 *Polyommatus (Agrodiaetus) peilei*	#07	**T**	**G**	**-**	**C**	**G**	**C**	**A**	**A**	**C**	**T**	**T**	**T**	**T**	**T**	**T**	**T**	**T**	**T**	**T**	**G**	**T**	**T**	**T**	**T**	**T**	**T**	**-**	**A**	**G**	**G**	**C**											
W136 *Polyommatus (Agrodiaetus) peilei*	#08	**A**	**A**	**-**	**-**	**-**	**-**	**A**	**A**	**T**	**-**	**-**	**-**	**-**	**-**	**-**	**-**	**-**	**-**	**-**	**-**	**-**	**-**	**-**	**-**	**-**	**-**	**-**	**A**	**A**	**G**	**C**											
W136 *Polyommatus (Agrodiaetus) peilei*	#09	**T**	**A**	**A**	**C**	**A**	**C**	**G**	**G**	**C**	**T**	**T**	**T**	**T**	**T**	**T**	**T**	**T**	**T**	**T**	**G**	**T**	**T**	**T**	**T**	**T**	**T**	**-**	**A**	**G**	**G**	**C**											
W136 *Polyommatus (Agrodiaetus) peilei*	#10	**T**	**G**	**-**	**C**	**G**	**C**	**A**	**A**	**T**	**T**	**T**	**T**	**T**	**T**	**T**	**T**	**-**	**-**	**T**	**G**	**T**	**T**	**T**	**T**	**T**	**-**	**C**	**G**	**G**	**G**	**C**											

		14	41	**128**	**131**	169	170	171	176	184	185	186	187	188	189	190	191	192	193	194	195	196	197	198	199	200	235	236	**326**	**336**	**337**	338	**345**	**346**	356								
W202 *Polyommatus (Agrodiaetus) peilei*	#01	**T**	**C**	**T**	**-**	**A**	**C**	**C**	**T**	**-**	**-**	**-**	**-**	**-**	**-**	**-**	**-**	**-**	**-**	**-**	**-**	**-**	**-**	**-**	**-**	**-**	**A**	**-**	**T**	**T**	**T**	**-**	**T**	**-**	**A**								
W202 *Polyommatus (Agrodiaetus) peilei*	#02	**T**	**T**	**T**	**A**	**A**	**C**	**C**	**T**	**T**	**C**	**G**	**C**	**G**	**T**	**C**	**G**	**G**	**C**	**G**	**A**	**C**	**G**	**T**	**G**	**C**	**G**	**G**	**C**	**T**	**T**	**T**	**T**	**-**	**A**								
W202 *Polyommatus (Agrodiaetus) peilei*	#03	**T**	**T**	**G**	**-**	**-**	**-**	**-**	**T**	**T**	**C**	**G**	**C**	**G**	**T**	**C**	**G**	**G**	**C**	**G**	**A**	**C**	**G**	**T**	**G**	**C**	**G**	**G**	**T**	**T**	**T**	**C**	**-**	**C**	**G**								
W202 *Polyommatus (Agrodiaetus) peilei*	#04	**T**	**T**	**T**	**-**	**A**	**C**	**C**	**C**	**-**	**-**	**-**	**-**	**-**	**-**	**-**	**-**	**-**	**-**	**-**	**-**	**-**	**-**	**-**	**-**	**-**	**A**	**-**	**T**	**T**	**-**	**C**	**-**	**C**	**G**								
W202 *Polyommatus (Agrodiaetus) peilei*	#05	**C**	**C**	**T**	**A**	**A**	**C**	**C**	**T**	**T**	**C**	**G**	**C**	**G**	**T**	**C**	**G**	**G**	**C**	**G**	**A**	**C**	**G**	**T**	**G**	**C**	**G**	**G**	**C**	**T**	**T**	**-**	**T**	**-**	**A**								
W202 *Polyommatus (Agrodiaetus) peilei*	#06	**T**	**T**	**T**	**A**	**A**	**C**	**C**	**T**	**T**	**C**	**G**	**C**	**G**	**T**	**C**	**G**	**G**	**C**	**G**	**A**	**C**	**G**	**T**	**G**	**C**	**G**	**G**	**C**	**T**	**T**	**T**	**T**	**-**	**A**								
W202 *Polyommatus (Agrodiaetus) peilei*	#07	**T**	**T**	**T**	**A**	**A**	**C**	**C**	**T**	**T**	**C**	**G**	**C**	**G**	**T**	**C**	**G**	**G**	**C**	**G**	**A**	**C**	**G**	**T**	**G**	**C**	**G**	**G**	**C**	**T**	**T**	**T**	**T**	**-**	**A**								
W202 *Polyommatus (Agrodiaetus) peilei*	#08	**T**	**C**	**T**	**-**	**A**	**C**	**C**	**T**	**-**	**-**	**-**	**-**	**-**	**-**	**-**	**-**	**-**	**-**	**-**	**-**	**-**	**-**	**-**	**-**	**-**	**A**	**-**	**T**	**-**	**-**	**C**	**-**	**C**	**G**								
W202 *Polyommatus (Agrodiaetus) peilei*	#09	**T**	**T**	**T**	**A**	**A**	**C**	**C**	**T**	**T**	**C**	**G**	**C**	**G**	**T**	**C**	**G**	**G**	**C**	**G**	**A**	**C**	**G**	**T**	**G**	**C**	**G**	**G**	**T**	**T**	**T**	**T**	**T**	**-**	**A**								
W202 *Polyommatus (Agrodiaetus) peilei*	#10	**T**	**C**	**T**	**-**	**A**	**C**	**C**	**T**	**T**	**C**	**G**	**C**	**G**	**T**	**C**	**G**	**G**	**C**	**G**	**A**	**C**	**G**	**T**	**G**	**C**	**A**	**-**	**T**	**T**	**T**	**C**	**-**	**C**	**G**								

		20	150	154	155	166	239	340	341	342																																	
V145 *Polyommatus (Agrodiaetus) karindus*	#01	**A**	**C**	**-**	**-**	**T**	**T**	**T**	**T**	**-**																																	
V145 *Polyommatus (Agrodiaetus) karindus*	#02	**A**	**C**	**-**	**-**	**T**	**T**	**T**	**-**	**-**																																	
V145 *Polyommatus (Agrodiaetus) karindus*	#03	**A**	**T**	**C**	**G**	**T**	**T**	**-**	**-**	**-**																																	
V145 *Polyommatus (Agrodiaetus) karindus*	#04	**A**	**T**	**C**	**G**	**T**	**T**	**-**	**-**	**-**																																	
V145 *Polyommatus (Agrodiaetus) karindus*	#05	**A**	**T**	**C**	**G**	**T**	**T**	**T**	**-**	**-**																																	
V145 *Polyommatus (Agrodiaetus) karindus*	#06	**A**	**C**	**-**	**-**	**T**	**C**	**-**	**-**	**-**																																	
V145 *Polyommatus (Agrodiaetus) karindus*	#07	**A**	**T**	**C**	**G**	**C**	**T**	**T**	**T**	**T**																																	
V145 *Polyommatus (Agrodiaetus) karindus*	#08	**A**	**C**	**-**	**-**	**T**	**T**	**T**	**-**	**-**																																	
V145 *Polyommatus (Agrodiaetus) karindus*	#09	**A**	**C**	**-**	**-**	**T**	**T**	**-**	**-**	**-**																																	
V145 *Polyommatus (Agrodiaetus) karindus*	#10	**G**	**C**	**-**	**-**	**T**	**T**	**-**	**-**	**-**																																	

		27	84	128	136	169	170	171	331	335	336	337	337	338	339	340	346	351	352	353	354																						
Z04 *Polyommatus (Agrodiaetus) karindus*	#01	**C**	**G**	**T**	**T**	**C**	**C**	**A**	**C**	**T**	**T**	**T**	**T**	**T**	**T**	**T**	**C**	**-**	**A**	**A**	**A**																						
Z04 *Polyommatus (Agrodiaetus) karindus*	#02	**C**	**G**	**T**	**T**	**C**	**C**	**A**	**T**	**T**	**T**	**T**	**T**	**T**	**T**	**T**	**C**	**-**	**A**	**A**	**A**																						
Z04 *Polyommatus (Agrodiaetus) karindus*	#03	**C**	**G**	**T**	**T**	**C**	**C**	**A**	**T**	**T**	**T**	**T**	**T**	**T**	**T**	**T**	**C**	**-**	**A**	**A**	**A**																						
Z04 *Polyommatus (Agrodiaetus) karindus*	#04	**C**	**G**	**T**	**T**	**C**	**C**	**A**	**T**	**T**	**T**	**T**	**T**	**T**	**T**	**T**	**C**	**-**	**A**	**A**	**A**																						
Z04 *Polyommatus (Agrodiaetus) karindus*	#05	**T**	**G**	**T**	**-**	**C**	**C**	**A**	**T**	**T**	**T**	**T**	**T**	**T**	**T**	**-**	**C**	**A**	**A**	**A**	**A**																						
Z04 *Polyommatus (Agrodiaetus) karindus*	#06	**T**	**G**	**T**	**-**	**C**	**C**	**A**	**T**	**T**	**T**	**T**	**T**	**T**	**T**	**-**	**C**	**A**	**A**	**A**	**A**																						
Z04 *Polyommatus (Agrodiaetus) karindus*	#07	**T**	**A**	**T**	**-**	**C**	**C**	**A**	**T**	**T**	**T**	**T**	**T**	**T**	**T**	**-**	**C**	**A**	**A**	**A**	**A**																						
Z04 *Polyommatus (Agrodiaetus) karindus*	#08	**C**	**G**	**T**	**T**	**C**	**C**	**A**	**T**	**T**	**T**	**T**	**T**	**T**	**T**	**-**	**C**	**-**	**A**	**A**	**A**																						
Z04 *Polyommatus (Agrodiaetus) karindus*	#09	**C**	**G**	**T**	**T**	**C**	**C**	**A**	**T**	**T**	**T**	**T**	**T**	**T**	**T**	**-**	**C**	**-**	**A**	**A**	**A**																						
Z04 *Polyommatus (Agrodiaetus) karindus*	#10	**T**	**G**	**G**	**-**	**-**	**-**	**-**	**C**	**-**	**-**	**-**	**-**	**-**	**-**	**-**	**-**	**-**	**-**	**-**	**-**																						

		7	38	43	79	127	128	148	171	172	173	229	301	318	319	320	321	322	323	324	325	326	327	328	329	330	331	332	333	334	347	348	349	350	352	353	354	355	357	358	391	400	472
W127 *Polyommatus (Agrodiaetus) morgani*	#01	**A**	**A**	**C**	**-**	**C**	**G**	**T**	**-**	**-**	**-**	**T**	**C**	**A**	**C**	**A**	**C**	**G**	**T**	**T**	**T**	**T**	**T**	**T**	**T**	**T**	**-**	**-**	**-**	**-**	**A**	**A**	**C**	**G**	**-**	**-**	**-**	**A**	**A**	**A**	**A**	**G**	**G**
W127 *Polyommatus (Agrodiaetus) morgani*	#02	**A**	**A**	**C**	**-**	**C**	**G**	**T**	**-**	**-**	**-**	**C**	**C**	**A**	**C**	**A**	**C**	**G**	**T**	**T**	**T**	**T**	**T**	**T**	**T**	**T**	**T**	**T**	**T**	**T**	**A**	**A**	**C**	**G**	**-**	**-**	**-**	**A**	**A**	**T**	**A**	**G**	**G**
W127 *Polyommatus (Agrodiaetus) morgani*	#03	**A**	**A**	**C**	**-**	**A**	**T**	**C**	**C**	**G**	**C**	**T**	**G**	**-**	**-**	**-**	**-**	**-**	**-**	**-**	**-**	**-**	**-**	**-**	**-**	**-**	**-**	**-**	**-**	**-**	**-**	**-**	**-**	**-**	**G**	**C**	**A**	**A**	**G**	**A**	**G**	**G**	**T**
W127 *Polyommatus (Agrodiaetus) morgani*	#04	**A**	**G**	**C**	**G**	**A**	**T**	**T**	**C**	**G**	**C**	**T**	**G**	**-**	**-**	**-**	**-**	**-**	**-**	**-**	**-**	**-**	**-**	**-**	**-**	**-**	**-**	**-**	**-**	**-**	**-**	**-**	**-**	**-**	**G**	**C**	**A**	**A**	**G**	**A**	**G**	**G**	**G**
W127 *Polyommatus (Agrodiaetus) morgani*	#05	**G**	**G**	**C**	**G**	**C**	**G**	**T**	**-**	**-**	**-**	**T**	**C**	**A**	**C**	**A**	**C**	**G**	**T**	**T**	**C**	**T**	**T**	**T**	**T**	**T**	**T**	**T**	**T**	**-**	**A**	**A**	**C**	**G**	**-**	**-**	**-**	**A**	**A**	**A**	**A**	**G**	**G**
W127 *Polyommatus (Agrodiaetus) morgani*	#06	**A**	**A**	**C**	**-**	**C**	**G**	**T**	**-**	**-**	**-**	**T**	**C**	**A**	**C**	**A**	**C**	**G**	**T**	**T**	**T**	**T**	**T**	**T**	**T**	**T**	**T**	**T**	**-**	**-**	**A**	**A**	**C**	**G**	**-**	**-**	**-**	**A**	**A**	**A**	**A**	**A**	**G**
W127 *Polyommatus (Agrodiaetus) morgani*	#07	**A**	**A**	**C**	**-**	**C**	**G**	**T**	**C**	**G**	**C**	**T**	**G**	**-**	**-**	**-**	**-**	**-**	**-**	**-**	**-**	**-**	**-**	**-**	**-**	**-**	**-**	**-**	**-**	**-**	**-**	**-**	**-**	**-**	**G**	**C**	**A**	**A**	**G**	**A**	**G**	**G**	**G**
W127 *Polyommatus (Agrodiaetus) morgani*	#08	**A**	**A**	**C**	**G**	**A**	**T**	**T**	**C**	**G**	**C**	**T**	**C**	**-**	**-**	**-**	**-**	**-**	**-**	**-**	**-**	**-**	**-**	**-**	**-**	**-**	**-**	**-**	**-**	**-**	**-**	**-**	**-**	**-**	**G**	**C**	**A**	**-**	**A**	**A**	**A**	**G**	**T**
W127 *Polyommatus (Agrodiaetus) morgani*	#09	**A**	**A**	**T**	**G**	**A**	**T**	**T**	**C**	**G**	**C**	**T**	**G**	**-**	**-**	**-**	**-**	**-**	**-**	**-**	**-**	**-**	**-**	**-**	**-**	**-**	**-**	**-**	**-**	**-**	**-**	**-**	**-**	**-**	**G**	**C**	**A**	**A**	**G**	**A**	**G**	**G**	**G**
W127 *Polyommatus (Agrodiaetus) morgani*	#10	**A**	**A**	**C**	**-**	**C**	**G**	**T**	**-**	**-**	**-**	**T**	**G**	**-**	**-**	**-**	**-**	**-**	**-**	**-**	**-**	**-**	**-**	**-**	**-**	**-**	**-**	**-**	**-**	**-**	**-**	**-**	**-**	**-**	**G**	**C**	**A**	**A**	**G**	**A**	**G**	**G**	**G**

**Table 2.** Uncorrected ‘‘p’’ distance matrix of clones.

Polyommatus (Agrodiaetus) peilei	W136_#01	W136_#02	W136_#03	W136_#04	W136_#05	W136_#06	W136_#07	W136_#08	W136_#09	W136_#10
W136_#01	-									
W136_#02	0.0019	-								
W136_#03	0.0196	0.0216	-							
W136_#04	0.0019	0.0039	0.0177	-						
W136_#05	0.0216	0.0236	0.0058	0.0196	-					
W136_#06	0.0058	0.0078	0.0176	0.0039	0.0196	-				
W136_#07	0.0117	0.0137	0.0157	0.0098	0.0098	0.0098	-			
W136_#08	0.0182	0.0202	0.0223	0.0162	0.0202	0.0162	0.0141	-		
W136_#09	0.0216	0.0236	0.0058	0.0196	0	0.0196	0.0098	0.0202	-	
W136_#10	0.0019	0.0039	0.0177	0	0.0196	0.0039	0.0098	0.0162	0.0196	-
**Average**	**0,0134**									
Polyommatus (Agrodiaetus) peilei	W202_#01	W202_#02	W202_#03	W202_#04	W202_#05	W202_#06	W202_#07	W202_#08	W202_#09	W202_#10
W202_#01	-									
W202_#02	0.0139	-								
W202_#03	0.0200	0.0157	-							
W202_#04	0.0142	0.022	0.0140	-						
W202_#05	0.0119	0.0059	0.0119	0.0259	-					
W202_#06	0.0139	0	0.0157	0.0219	0.0059	-				
W202_#07	0.0139	0	0.0157	0.0219	0.0059	0	-			
W202_#08	0.0122	0.0239	0.0160	0.0061	0.0239	0.0239	0.0239	-		
W202_#09	0.0119	0.0019	0.0137	0.0199	0.0078	0.0019	0.0019	0.0219	-	
W202_#10	0.0100	0.0176	0.0177	0.0080	0.0176	0.0176	0.0176	0.0060	0.0157	-
**Average**	**0,0135**									
Polyommatus (Agrodiaetus) karindus	V145_#01	V145_#02	V145_#03	V145_#04	V145_#05	V145_#06	V145_#07	V145_#08	V145_#09	V145_#10
V145_#01	-									
V145_#02	0.0019	-								
V145_#03	0.0078	0.0059	-							
V145_#04	0.0078	0.0059	0	-						
V145_#05	0.0059	0.0039	0.0019	0.0019	-					
V145_#06	0.0059	0.0059	0.0078	0.0059	0.0078	-				
V145_#07	0.0078	0.0098	0.0078	0.0078	0.0078	0.0137	-			
V145_#08	0.0019	0	0.0059	0.0059	0.0039	0.0059	0.0098	-		
V145_#09	0.0039	0.0019	0.0039	0.0039	0.0059	0.0019	0.0117	0.0019	-	
V145_#10	0.0059	0.0039	0.0078	0.0059	0.0078	0.0039	0.0137	0.0039	0.0019	-
**Average**	**0,0056**									
Polyommatus (Agrodiaetus) karindus	Z704_#01	Z704_#02	Z704_#03	Z704_#04	Z704_#05	Z704_#06	Z704_#07	Z704_#08	Z704_#09	Z704_#10
Z704_#01	-									
Z704_#02	0.0019	-								
Z704_#03	0.0019	0	-							
Z704_#04	0.0019	0	0	-						
Z704_#05	0.0098	0.0078	0.0078	0.0078	-					
Z704_#06	0.0098	0.0078	0.0078	0.0078	0	-				
Z704_#07	0.0117	0.0098	0.0098	0.0098	0.0019	0.0019	-			
Z704_#08	0.0039	0.0019	0.0019	0.0019	0.0059	0.0059	0.0078	-		
Z704_#09	0.0039	0.0019	0.0019	0.0019	0.0059	0.0059	0.0078	0	-	
Z704_#10	0.0141	0.0162	0.0162	0.0162	0.0162	0.0162	0.0182	0.0182	0.0182	-
**Average**	**0,0072**									
Polyommatus (Agrodiaetus) morgani	V127_#01	V127_#02	V127_#03	V127_#04	V127_#05	V127_#06	V127_#07	V127_#08	V127_#09	V127_#10
V127_#01	-									
V127_#02	0.0101	-								
V127_#03	0.0289	0.0307	-							
V127_#04	0.0267	0.0328	0.0083	-						
V127_#05	0.0122	0.0141	0.0368	0.0246	-					
V127_#06	0.0041	0.0101	0.0267	0.0205	0.0121	-				
V127_#07	0.0144	0.0246	0.0083	0.0083	0.0266	0.0185	-			
V127_#08	0.0165	0.0266	0.0104	0.0104	0.0246	0.0246	0.0146	-		
V127_#09	0.0226	0.0328	0.0083	0.0041	0.0287	0.0267	0.0083	0.0104	-	
V127_#10	0.0124	0.0226	0.0104	0.0104	0.0267	0.0165	0.0021	0.0167	0.0104	-
**Average**	**0,0177**									

There were 11 single-base substitutions, 3 mono and 4 multi-nucleotide indels, in clones of specimen W136 (Polyommatus (Agrodiaetus) peilei). Interestingly, that clone “W136_#08” differed significantly from all others in having 16-nycleotide polyT deletion at positions “329-344” and 3 base indel at position “171-173”. Clones of second specimen Polyommatus (Agrodiaetus) peilei (W202) had 8 sites with single nucleotide substitutions and 8 positions, where mono multi-nucleotide indels occurred. Three clones had large polymorphic 17-nucleotide indel at positions “184-200”. Variation among clones was significant, with intragenomic differences ranging from 0.0% to 2.39%. The average intragenomic genetic distances for two specimens of Polyommatus (Agrodiaetus) peilei (W136 and W202) were very similar: 1.34% and 1.35% respectively.

Polyommatus (Agrodiaetus) karindus had significantly lower rate of intragenomic variability. Specimens V145 and Z704 had 9 and 10 polymorphic positions, respectively. Furthermore, majority number of indels and base substitutions of Z704 specimen is accounted for by one clone (Z704#10). It has one single substitution and 3 multi-nucleotide deletions, which never occurred in other clones. The average intragenomic genetic distances for two specimens of Polyommatus (Agrodiaetus) karindus (V145 and Z704) were: 0.56% and 0.72%, respectively. The highest value was 1.82%.

Clones of *Polyommatus (Agrodiaetus) morgani ITS2* showed greater diversity than the other 2 species. For instance, the genetic distance between V127#05 clone and V127#03 was 3.68%. The average intragenomic genetic distance was also significantly higher for this species – 1.77%

**Figure 2.**
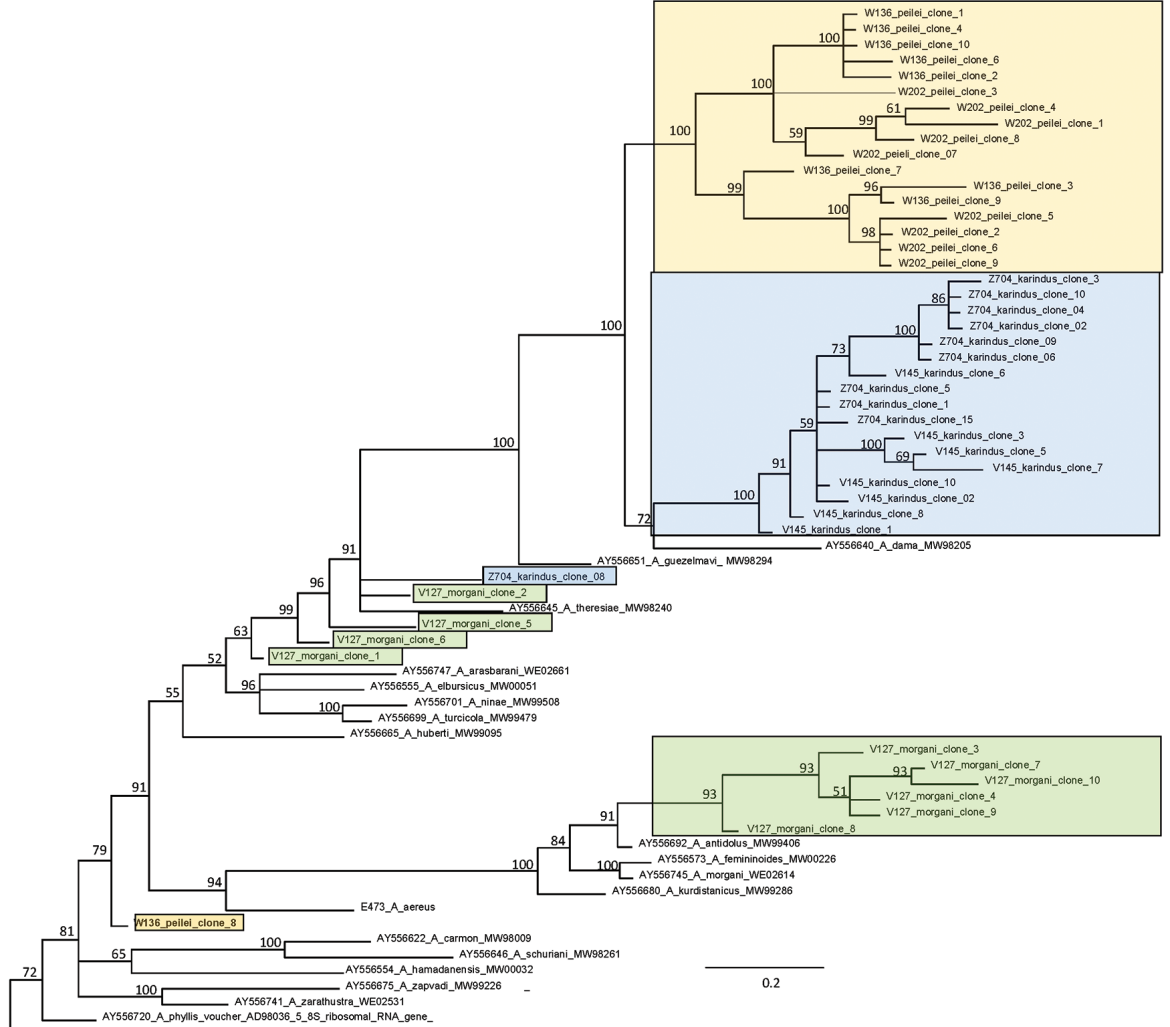
Fragment of consensus Bayesian tree of the subgenus *Agrodiaetus* inferred from *ITS2* sequences. Posterior probability values >50% are shown. The complete tree is given online in the Suppl. material 1. Cloned sequences of three studied species are highlighted: Polyommatus (Agrodiaetus) peilei – orange colour, Polyommatus (Agrodiaetus) karindus – blue colour, Polyommatus (Agrodiaetus) morgani – green colour.

In Bayesian analysis 50 cloned amplicons from Polyommatus (Agrodiaetus) peilei, Polyommatus (Agrodiaetus) karindus, Polyommatus (Agrodiaetus) morgani and *ITS2* sequences from all *Agrodiaetus* species available in the GenBank were included, giving a total of 127 sequences. Since *Polyommatus icarus* (Rottemburg, 1775) was earlier inferred as sister clade to the subgenus *Agrodiaetus* (Talavera et al. 2013), we used one specimen (GenBank accession number AY556732) as outgroup to root the phylogeny. Fragment of consensus Bayesian tree, showing clusterization of cloned sequences is given in Figure 2. The complete tree is given online in the Suppl. material 1.

## Discussion

Despite the popularity of the *ITS2* nuclear rDNA marker in systematics of different groups of animals and plants, its variability on intraspecific and intraindividual level is still poorly known. The occurrence of multiple *ITS2* copies within a single genome should be accounted for before rDNA is used for phylogenetic or population studies. Furthermore, investigation of rates of intra-individual polymorphism can lighten addressing questions regarding speciation, species hybridization end evolutionary history. It is generally considered that multigene families, such as rDNA maintain homogeneity of all copies as a result of concerted evolution (processes of gene conversion and unequal crossing over) (Zimmer et al. 1980, Dover 1982). Mutations rapidly spread to all members of the gene family even if there are arrays located on different chromosomes (Dover 1982, Arnheim1983, Gerbi 1985, Tautz et al. 1988). The efficiency of homogenization of rDNA is usually high (Liao 1999). Concerted evolution of noncoding sequences, such as internal transcribed spacers, can result in fixed interspecific differences and intraspecific homogeneity. Despite this assumption, our results show, that intraindividual variability can be maintained, when mutation rates are higher than rates of homogenization. This can lead to erroneous phylogenetic reconstructions and species misidentification.

Here we contribute with the first insight into the intraspecific *ITS2* diversity in the blue butterflies of subgenus *Agrodiaetus*.

The *ITS2* of all specimens of three *Agrodiaetus* species - (Polyommatus (Agrodiaetus) peilei, Polyommatus (Agrodiaetus) karindus and Polyommatus (Agrodiaetus) morgani) were intragenomically variable. There were a number of indels and base substitutions accounting for both the length and sequence variabilities. Numerous indels lead to length variation (477-512 bp) of studied sequences. Bayesian phylogenetic reconstruction revealed that cloned sequences of certain individuals did not form a monophyletic unanimity, but the majority of clones clustered together within species borders. In particular, clones of Polyommatus (Agrodiaetus) peilei and Polyommatus (Agrodiaetus) karindus individuals are recovered as two distinct separated clusters, both with a Bayesian posterior probability of 1.00. The position of 6 clones of Polyommatus (Agrodiaetus) morgani specimen on the *ITS2* tree support the conclusion that abovementioned species belong to “*antidolus*” species-group which comprise 5 allopatric in distribution, closely related taxa: Polyommatus (Agrodiaetus) femininoides (Eckweiler, 1987), Polyommatus (Agrodiaetus) antidolus (Rebel, 1901), Polyommatus (Agrodiaetus) aereus (Eckweiler, 1998), Polyommatus (Agrodiaetus) kurdistanicus (Forster, 1961) and Polyommatus (Agrodiaetus) morgani. “*Antidolus*” clade revealed with a high level of posterior probability. However, when considering all cloning data, in some cases differences between cloned sequences of the same individual were greater than that between species. For instance, the remainder of Polyommatus (Agrodiaetus) morgani clones are placed as the basal taxa to clade, consist of Polyommatus (Agrodiaetus) guezelmavi (Olivier, Puplesiene, van der Poorten, De Prins & Wiemers, 1999), Polyommatus (Agrodiaetus) dama (Staudinger, 1892) and majority of Polyommatus (Agrodiaetus) peilei and Polyommatus (Agrodiaetus) karindus clones. One clone of Polyommatus (Agrodiaetus) karindus (Z704_#08) also was recovered as sister taxa to abovementioned clade. Finally, W136_#08 clone of Polyommatus (Agrodiaetus) peilei is found to be more genetically distant from other clones of this individual than the great number of other species of the subgenus *Agrodiaetus* (Figure 2).

Recent works showed that tandem arrays of rRNA genes in most Lepidoteran species form one or two so-called rDNA clusters, although some exceptions in cluster number exist (Nguen et al. 2010). Data on the number and distribution of rDNA clusters in genomes of lycaenid butterflies are very scarce. Previous investigation by Vershinina et. al. (2015) examined ribosomal clusters in seven blue butterflies of the genus *Polyommatus* and showed the presence of two different variants of the location of major rDNA clusters in *Polyommatus* species: with one or two rDNA-carrying chromosomes in haploid karyotype (Vershinina et al. 2015). Polyommatus (Agrodiaetus) peilei, Polyommatus (Agrodiaetus) karindus and Polyommatus (Agrodiaetus) morgani were among studied species, which bear a single rDNA cluster. Thus, all intragenomic *ITS2* patterns for every individual analysed, belong to a single rDNA cluster, which means that examined level of intragenomic variability not caused by sequencing *ITS2* copies located on different chromosomes.

To conclude, our study demonstrates that the results of direct sequencing may not describe the actual and entire set of sequence variants. Level of divergence between clones of one individual can be comparable to interspecific genetic differences variations or even exceed them. Hence, cloning and subsequent intraindividual haplotypes handling are required for reliable phylogenetic reconstructions.

## Supplementary materials

Supplementary material 1 Consensus Bayesian tree of the subgenus Polyommatus (Agrodiaetus) inferred from ITS2 sequences Data type: TIFF image Explanation note: Consensus Bayesian tree of the subgenus Polyommatus (Agrodiaetus) inferred from ITS2 sequences. Posterior probability values >50% are shown. This dataset is made available under the Open Database License (http://opendatacommons.org/licenses/odbl/1.0/). The Open Database License (ODbL) is a license agreement intended to allow users to freely share, modify, and use this Dataset while maintaining this same freedom for others, provided that the original source and author(s) are credited.Nazar A. Shapoval, Vladimir A. Lukhtanov
