# Supplementary figures and images for: Intragenomic variations of multicopy ITS2 marker in Agrodiaetus blue butterflies (Lepidoptera, Lycaenidae)
Source: Comp Cytogenet. 2015 Aug 7;9(4):483–97. doi: 10.3897/CompCytogen.v9i4.5429 (PMC4698565; doi:10.3897/CompCytogen.v9i4.5429)

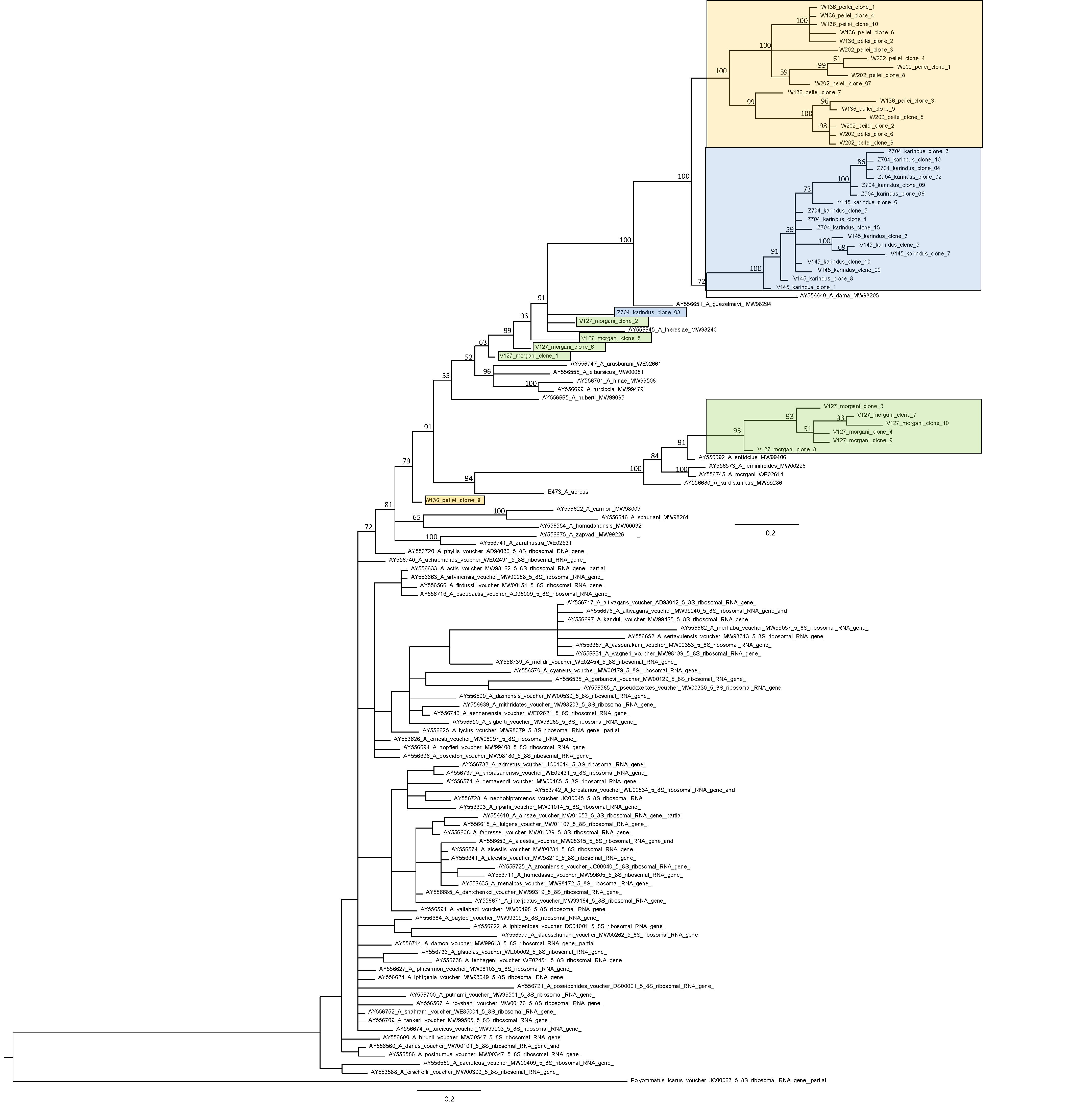

Supplement: Supplementary material 1 — Consensus Bayesian tree of the subgenus Polyommatus (Agrodiaetus) inferred from ITS2 sequences [file CompCytogen-9-483-s001.tiff]
